# Exercise Capacity and Cardiorespiratory Fitness in Children with Congenital Heart Diseases: A Proposal for an Adapted NYHA Classification

**DOI:** 10.3390/ijerph19105907

**Published:** 2022-05-12

**Authors:** Daniel Neunhaeuserer, Francesca Battista, Barbara Mazzucato, Marco Vecchiato, Giulia Meneguzzo, Giulia Quinto, Josef Niebauer, Andrea Gasperetti, Vladimiro Vida, Giovanni Di Salvo, Maurizio Varnier, Andrea Ermolao

**Affiliations:** 1 Sports and Exercise Medicine Division, Department of Medicine, University of Padova, Via Giustiniani 2, 35128 Padova, Italy; daniel.neunhaeuserer@unipd.it (D.N.); barbara.mazzucato.9178@gmail.com (B.M.); marcovecchiato.md@gmail.com (M.V.); giulia.meneguzzo.gm@gmail.com (G.M.); giulia.quinto9@gmail.com (G.Q.); a.gasperetti@libero.it (A.G.); varniermedsport@gmail.com (M.V.); andrea.ermolao@unipd.it (A.E.); 2 Clinical Network of Sports and Exercise Medicine of the Veneto Region, 35131 Padova, Italy; 3 Institute of Sports Medicine, Prevention and Rehabilitation, Paracelsus Medical University of Salzburg, Lindhofstraße 20, 5020 Salzburg, Austria; j.niebauer@salk.at; 4 Pediatric and Congenital Cardiac Surgery, Department of Cardio-Thoracic-Vascular Sciences and Public Health, University of Padova, Via Giustiniani 2, 35128 Padova, Italy; vladimiro.vida@unipd.it; 5 Pediatric Cardiology Unit, Department of Woman and Child’s Health, University of Padova, Via Giustiniani 2, 35128 Padova, Italy; giovanni.disalvo@unipd.it

**Keywords:** cardiopulmonary exercise test, New York Heart Association classification, functional evaluation, physical activity

## Abstract

Objective: To propose and evaluate an adapted NYHA classification for children with congenital heart disease (CHD) as a feasible clinical tool for classifying patients’ fitness, cardiorespiratory efficiency and functional limitations during their ordinary daily activities, which are also characterized by vigorous and competitive physical exercise among peers. Methods: This cross-sectional investigation analyzed 332 patients (13.1 ± 3.01 y/o) who underwent surgical repair of CHD and performed Cardiopulmonary Exercise Testing (CPET). Patients were divided into NYHA class I, IIA and IIB by specific questioning regarding functional limitation and performance compared to peers and at strenuous intensity. Class IIA was characterized by slight exercise limitation only for strenuous/competitive activities, whereas IIB for already ordinary physical activities. These NYHA classes were compared with maximal CPET on treadmill. Results: Patients’ exercise capacity (exercise time, METs), aerobic capacity (VO_2_peak) and chronotropic response were found progressively impaired when NYHA class I was compared with IIA and IIB. Indeed, ventilatory-perfusion mismatch (PETCO_2_, VE/VCO_2_) significantly worsened from NYHA class I to IIA, while no difference was found between IIA and IIB. Conclusion: This adapted NYHA-CHD classification could allow regular functional evaluations and accurate assessments by clinicians, leading to facilitated clinical management and timely medical interventions.

## 1. Introduction

The estimated prevalence of congenital heart disease (CHD) at birth is 0.8% [1]. The overall survival of these patients has greatly increased over the years [1,2,3,4], leading to a special population of patients with CHD who require specific medical care, particularly regarding the maintenance of their functional capacity and thus quality of life [5,6]. As has already been reported in the literature, many of these patients are asymptomatic at rest and during low-intensity exercise, but do experience physical limitation at higher efforts [3]. Such physical impairment could be uncovered by detailed medical history assessment and objectively measured with Cardiopulmonary Exercise Testing (CPET) [7,8]. Since CPET is not easily available for all patients with CHD and can generally not be performed on a regular basis, the clinical functional categorization by the symptom-guided New York Heart Association (NYHA) classification is widely used, also in children [9,10,11]. However, the NYHA classification was developed for the evaluation of heart failure in mainly older and/or comorbid patients [9,10], who rarely engage in physical activities beyond the ordinary activities of daily living [4,11], which is in evident contrast to patients with CHD, who tend to be more physically active and often participate in organized sports (i.e., basketball or soccer matches) [8,12]. 

A more accurate medical history evaluation focused on exercise related symptoms may be needed, since most of these patients are asymptomatic at rest and during exercise at light to moderate intensities; the original NYHA classification, particularly concerning classes I and II, may not be sufficiently sensitive for this specific population, where functional limitation is frequently underestimated [13,14]. The standard NYHA classification is thus not useful for young patients with CHD because of different ordinary habits, exercise tolerance and engagement also in competitive physical activities and exercise among peers during leisure time activities and sports. Indeed, children’s activities of daily living are different from those of adults and include strenuous physical exercise while playing. However, a comparably simple functional evaluation method currently does not exist for this increasing pediatric population but would be strongly needed for clinical routine of pediatricians and clinicians in charge of these patients. Moreover, a feasible and reproducible functional evaluation tool for all healthcare professionals dealing with CHD could improve care planning in terms of baseline screening but also to monitor patients’ follow-up. The regular medical history evaluation of patients’ functional capacity, thereby indirectly estimating their cardiorespiratory fitness, could lead to a timely referral to more specific evaluations and is also useful to provide counseling regarding sports eligibility, type of exercise and ordinary physical activities.

The aim of our study was to propose a clinically useful and feasible functional evaluation tool by adapting the NYHA classification for young patients with CHD, which may better reflect patients’ functional limitation, physical fitness, and cardiorespiratory efficiency.

## 2. Materials and Methods

This study was designed as a retrospective cross-sectional investigation in 332 young patients who underwent surgical repair of CHD in their childhood and performed a CPET for functional evaluation between 2004 and 2018 at the Sports and Exercise Medicine Division of the Department of Medicine, University of Padova (Italy).

All these patients were referred to our Centre from the Pediatric Cardiology Unit of the Department of Women’s and Children’s Health, University of Padova, following an institutional diagnostic–therapeutic pathway of clinical assistance. Moreover, the presented observational study outcomes were gained from routinely performed clinical assessments over years. Indeed, after obtaining written informed consent, the evaluations and CPET were carried out as multidisciplinary follow-up of these patients. The study was conducted in accordance with the Declaration of Helsinki and has been approved by the Ethics Committee for Clinical Research of Padova (95n/AO/21). Subjects surgically treated due to coarctation of the aorta, transposition of the great arteries, tetralogy of Fallot, functionally univentricular heart corrected with Fontan procedure, and other complex heart anomalies were included in this clinical trial. Moreover, similarly to the approach proposed by the EAPC and ESC for adolescents with CHD, the study population was analyzed based on functional parameters, rather than focusing on specific anatomical defects [8]. Patients older than 18 years of age and those who could not perform CPET were excluded. Furthermore, subjects classified as NYHA III or IV were not considered for this investigation.

### 2.1. Functional Classification

The NYHA functional class was determined by a physician based on the assessment of patients’ self-reported symptoms at the time of CPET: all patients were divided into the two main functional classes, i.e., NYHA I (no limitation of physical activity) and NYHA II (asymptomatic at rest, slight limitation of physical activity). However, a further medical history evaluation was performed with the following specific additional questions, in order to screen for those patients who might underestimate the presence of functional limitations:

- Do you feel you are limited during physical activities or exercise compared to your peers?
- Would an external viewer notice some differences in your performance compared to your peers or teammates?
- Does it happen that you feel any kind of functional limitation during vigorous physical activities or exercise (i.e., physical activities or exercise where the intensity is such that it is impossible to speak—RPE 18/20 on Borg scale)?

Patients were classified to NYHA I if a negative response was given to all questions, otherwise they were added to NYHA class II. Moreover, NYHA class IIA is characterized by slight exercise limitation only for strenuous or competitive exercise, whereas NYHA class IIB by already ordinary physical activities causing fatigue, palpitations, dyspnea, or thoracic pain. With regard to the definition of “ordinary activities”, it is important to consider that a pediatric population with CHD was studied. Indeed, children are rarely sedentary individuals and spend much more time in playing and moving around; thus, “ordinary activity” is usually much more strenuous when compared to that of adults.

### 2.2. Cardiopulmonary Exercise Testing

Standardized CPET was performed, assessing patients’ aerobic and exercise capacity as well as cardiorespiratory efficiency by an incremental test protocol on treadmill, conducting the Bruce Ramp protocol (Jaeger-Masterscreen-CPX, Carefusion, Germany). However, one patient performed the modified Bruce Ramp protocol [15]. All these incremental exercise tests were performed until patients’ exhaustion. The CPET was considered maximal when patients reached a Borg rating of perceived exertion (RPE) ≥18/20 or a heart rate max (HRmax) >85% of the predicted HR for age or a RER ≥1.10. Other criteria for test discontinuation were the onset of symptoms (chest pain, heart palpitation, dyspnea, dizziness, joint-muscle pain), repetitive complex arrhythmias, and abnormal arterial blood pressure responses (hypertensive response to exercise or systolic blood pressure values decreasing by more than 20 mmHg from baseline). Ventilatory and gas exchange measurements were sampled breath by breath to assess oxygen consumption (VO_2_), minute ventilation (VE), carbon dioxide output (VCO_2_) and the associated CPET parameters. The oxygen uptake efficiency slope (OUES) was calculated as the coefficient of the linear relationship between VO_2_ and the logarithm of VE [16]. Arterial blood pressure, ECG and peripheral oxygen saturation were continuously monitored at rest, during incremental exercise and until the sixth minute of the recovery phase.

### 2.3. Statistical Analyses

Mean value with standard deviation (SD) or median with interquartile range (IQR) were used to describe continuous variables, when respectively normally or not-normally distributed. All variables were tested for normality using the Shapiro–Wilk test and based on their distribution, the differences among groups were evaluated using a parametric one-way ANOVA or a non-parametric Kruskal–Wallis test. Bonferroni or Dunn’s multiple comparison test were used for post-hoc analyses. For categorical variables Chi square test was performed. All p values were two-sided, considering <0.05 as statistically significant. Data were analyzed using SPSS 20.0 for Windows (SPSS Inc., Chicago, IL, USA).

## 3. Results

The study population was composed of 332 patients (66.3% males) with a mean age of 13.1 ± 3.01 years and a BMI of 19.0 ± 3.3 kg/m^2^. They were distributed as follows with regard to the different congenital heart defects: coarctation of the aorta (*n* = 121, 36.4%), tetralogy of Fallot (*n* = 77, 23.2%), functionally univentricular hearts who underwent a Fontan intervention (*n* = 65, 19.6%), transposition of the great arteries (*n*= 48, 14.4%), complete atrio-ventricular septal defect (*n* = 6, 1.8%), transposition of the great arteries associated with a ventricular septal defect (*n* = 5, 1.5%), congenitally corrected transposition of the great arteries (*n* = 4, 1.2%), complex anomalies (*n* = 4, 1.2%), and partial atrio-ventricular septal defect (*n* = 2, 0.6%) (Supplementary Table S1).

Patients’ mean maximal exercise and aerobic capacity was characterized by 15.6 ± 2.7 metabolic equivalents of task (METs) and a VO_2_peak of 38.3 ± 9.0 mL·min-1·kg-1, reaching a maximal heart rate (HR) of 87.3 ± 9.4% of predicted.

The patients were distributed according to the respective functional classes as follows: 235 patients (70.8%) were located in NYHA class I, 66 (19.9%) in NYHA class IIA and 31 (9.3%) in NYHA class IIB. Data shown in Table 1 demonstrate a significant difference regarding exercise and aerobic capacity when comparing patients classified as NYHA class I with those of both NYHA classes IIA and IIB (exercise time, METs, VO_2_peak; all *p* < 0.001). Patients’ chronotropic response to exercise also differed significantly between these classes (maximal HR, percentage of maximal predicted HR, HR Reserve; all *p* < 0.001). Furthermore, CPET parameters evaluating cardiorespiratory efficiency and ventilatory-perfusion mismatching were found significantly better in patients in NYHA class I when compared to those in classes IIA and IIB (oxygen saturation at rest and at peak exercise, VE/VCO_2_ and PETCO_2_ at anaerobic threshold; all *p* <0.001). This was also confirmed by the OUES (*p* < 0.001), a parameter evaluating cardiorespiratory efficiency and fitness, which does less depend on patients’ compliance and maximal exercise intensities.

**Figure 1:**
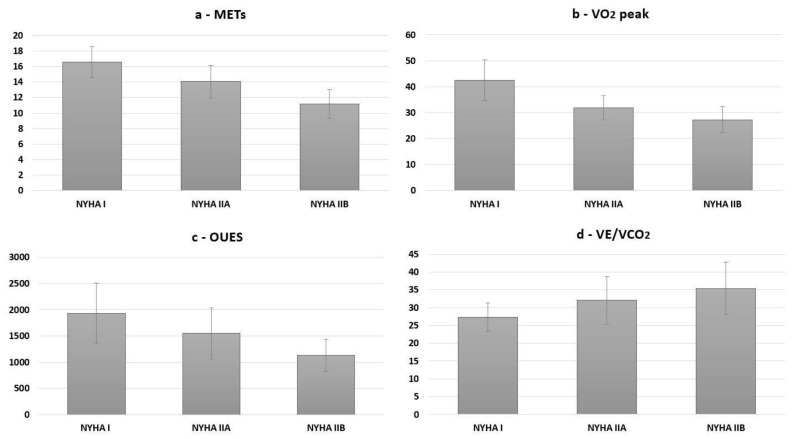
Functional and aerobic capacity as well as cardiorespiratory efficiency according to adapted NYHA-CHD classes. (**a**) shows maximal metabolic equivalents of task (METs), (**b**) shows peak oxygen consumption (VO_2_ peak; mL·kg-1·min-1), (**c**) shows the Oxygen Uptake Efficiency Slope (OUES; mL·log-l), and (**d**) shows the VE/VCO_2_ at the anaerobic threshold. These CPET markers show a progressive degree of functional limitation and worsening of cardiorespiratory efficiency as the NYHA-CHD class increases. Data are presented as mean with standard deviation.

Comparing patients in NYHA class IIA with those in IIB, the latter demonstrated significantly lower functional capacity (exercise time, METs; both *p* < 0.001) and aerobic power (VO_2_peak *p* = 0.010). With regard to cardiorespiratory efficiency, the OUES differed significantly between both NYHA class II subgroups, resulting markedly lower in the class IIB (*p* = 0.001; Figure 1). Other parameters evaluating ventilatory-perfusion mismatch, like peak oxygen saturation, PETCO_2_ and VE/VCO_2_ at the anaerobic threshold, showed similar aggravating trends of the absolute values without reaching a statistically significant difference between NYHA class IIA and IIB.

## 4. Discussion

The standard NYHA classification, as a functional evaluation tool, is difficult to apply to children with corrected CHD, since exercise tolerance is higher and daily requirements are different. An appropriate, simple, and reproducible functional classification is currently lacking for this specific population, but would be needed in different healthcare settings for screening, follow-up and clinical decision making, particularly during patients’ physical development [6]. Disease progression or worsening of hemodynamic alterations could lead to premature functional limitations and reduced exercise tolerance, which should thus be regularly assessed. A timely referral to more specific medical evaluations could thereby be achieved. Indeed, our study has demonstrated a progressive worsening of exercise capacity and tolerance as well as aerobic power with the increase in NYHA class from I to IIA and IIB. A similar gradual deterioration was found for parameters reflecting cardiorespiratory efficiency and ventilatory-perfusion mismatching. Thus, data revealed that it is indeed feasible to further sub-classify NYHA classes by specific questioning, which leads to objectively different subgroups with regard to exercise limitations, physical fitness and cardiorespiratory efficiency, known important prognostic markers in health and disease [17]. This clinically feasible and non-invasive assessment may become a useful method for evaluating our young patients’ real functional status and future prognosis. The adapted “NYHA-CHD” classification can thus be proposed as symptom-guided clinical tool for young patients with CHD, which is just what the standard NYHA classification represents for adults with heart failure [18].

It is already known from different previous studies that adults with CHD have a reduced exercise and aerobic capacity when compared to healthy controls [3,7,13,14,19,20,21,22,23,24]. Moreover, Diller et al. [14] demonstrated that exercise capacity gradually declines with worsening functional classes. Indeed, it was also emphasized how the NYHA classification helps to distinguish patients with mild from those with moderate or severe functional impairment [25]. However, authors also concluded that the NYHA class may underestimate the true grade of exercise limitation in a population with CHD [20,24]. Some patients with CHD, in fact, despite having a depressed cardiac function, do not always seem to realize their physical limitations, maybe because they adjust their physical activity level to their clinical situation [4,26]. Thus, it was suggested that the application of a strictly subjective classification might not be useful to precisely determine the real degree of exercise intolerance in this population. However, Gavotto et al. reported that VO_2_peak was strongly associated with the NYHA functional class in patients with a systemic right ventricle [24]. Indeed, the NYHA class seemed to be the strongest predictor of maximal exercise capacity in this cohort.

Our study confirmed a progressive worsening of cardiorespiratory fitness with the increase in NYHA classes. In particular, our analyses highlighted two distinctively different subgroups of patients within the second stage of the NYHA classification, i.e., NYHA class IIA and IIB. NYHA class I included patients who were totally asymptomatic even during strenuous and competitive physical activities; they represented the large majority of our population (70.8%) and showed significantly higher exercise capacity. NYHA class II, when subdivided into IIA and IIB, showed that NYHA class IIA comprised young patients who were asymptomatic during ordinary physical activities but had slight functional limitations during high-intensity physical efforts, while NYHA class IIB patients were classified as those who were asymptomatic at rest but who already had slight functional limitations during ordinary physical activities, resulting in fatigue, palpitations, dyspnea or thoracic pain [27]. The proposed simple additional questions with regard to patients’ exercise tolerance were able to identify patients who commonly underestimated their exercise limitations, but also those who overestimated their functional capabilities (Table 2).

Indeed, the NYHA subgroups IIA and, even more, IIB, had a significantly lower functional/aerobic capacity and exercise tolerance compared to their peers in NYHA class I. Thus, our data suggest that the traditional NYHA classification could be split into two subgroups in order to adequately characterize the broad spectrum of young patients grown up with CHD (NYHA-CHD). This aspect is of clinical importance, since limitations at higher exercise intensities or while playing with peers during physical leisure time activities, sports or competitive exercise may suggest significant cardiorespiratory and/or hemodynamic alterations, which affect ordinary activities of daily living and thus quality of life of these young patients [28]. Furthermore, a degradation in time in functional class will provide useful clinical information and lead to a timely referral to more specific evaluations by medical specialists [29]. Thus, such a simple functional assessment tool might be useful for all clinicians and exercise professionals in charge of the management of this increasing number of patients [27]. 

Additionally, CPET parameters analyzing submaximal exercise capacity, chronotropic response to exercise and cardiorespiratory efficiency provided similar results. Moreover, a progressive deterioration of the OUES, peak oxygen saturation, PETCO_2_ and VE/VCO_2_ were shown with increasing NYHA class, which was particularly apparent when comparing class I with IIA. Even though these parameters, reflecting ventilatory-perfusion mismatch, also worsened in absolute terms and with a similar trend when NYHA class IIA was compared with IIB, statistical significance was only partially reached, probably due to the lower sample size in NYHA class IIB (Figure 1). However, it was shown that the functional limitation displayed by a NYHA class IIB is mirrored by an effective worsening in exercise capacity, tolerance and cardiorespiratory efficiency, also when compared to NYHA class IIA patients. Indeed, this seems also partly to be reflected by an increase in ventilatory-perfusion mismatch, which becomes clinically relevant in NYHA class IIB patients, considering their average values of peak oxygen saturation, OUES, PETCO_2_ and VE/VCO_2_ at the anaerobic threshold. Indeed, these parameters have also been proposed as prognostic markers for patients with heart failure, and should thus also be regularly evaluated in this population, as well as in adults with CHD [30]. Nevertheless, since CPET is not always easily available, our study showed that patients can also be classified by simple specific questioning, thereby adapting the NYHA classification for this population. Moreover, certain exercise induced cardiorespiratory alterations may present only at vigorous intensities, which emphasizes the importance of evaluating specifically competitive physical exercise among peers and sports-related playing activities, representing ordinary habits in this population. Thus, the proposed modified NYHA-CHD classification for young patients with CHD could provide a feasible method for routinely evaluating clinical disease severity, which might lead to a timely referral to more specific evaluations and subsequently to medical and surgical treatment adaptations [6]. This hypothetical application should be investigated in future prospective long-term trials to further evaluate the clinical utility of this specific NYHA-CHD classification.

### 4.1. Limitations

Since the underlying type of CHD may have influenced the allocation to NYHA classes, future trials may focus on populations with specific heart defects. Indeed, in the NYHA classes IIA and IIB, the majority of patients were those with functionally univentricular heart corrected with Fontan procedure, tetralogy of Fallot and complex anomalies, while coarctation of the aorta predominated in NYHA class I. However, the recommendations for physical activity in adolescents with CHD by the EAPC and ESC are also based on common hemodynamic and electrophysiological parameters, rather than focusing on specific anatomical defects [8]. Moreover, the proposed NYHA-CHD classification has to be evaluated with a healthy and age-matched control population as well as in a population in which patients in functional class NYHA III-IV are not excluded. Further studies with more selective populations and including patients without need for surgical repair could also be useful to provide additional information regarding the validity of the NYHA-CHD classification. Furthermore, the role of ventilatory-perfusion mismatch as the main limiting factor of functional capacity and exercise tolerance in patients with CHD should be investigated.

### 4.2. Perspectives

The overall survival of patients with CHD has greatly increased in recent decades, and many of them engage in leisure time physical activities, exercise and sports. This population requires specific professional support and regular follow-up to guarantee the long-term maintenance of good clinical conditions, functional capacity and thus quality of life [4]. This is particularly true for patients with surgically corrected CHD, since the long-term adaptations related to exercise interventions should be monitored and investigated, in order to reduce the risk of cardiovascular adverse events during sports and provide more evidence on the outcomes. CPET may represent one of the most important functional evaluations for these patients in order to unmask alterations of cardiorespiratory efficiency but also to provide a prognostic risk stratification needed for precocious treatment and training adaptations. However, CPET might not be regularly and easily available for most patients. A feasible, reproducible, and economical evaluation tool using standardized medical history questioning makes it possible to functionally categorize these patients according to a specifically modified NYHA-CHD classification, which adequately reflects patients’ exercise capacity and cardiorespiratory efficiency. The adapted NYHA-CHD classification may refine the clinical and functional evaluation for all professionals working with these patients, to improve care planning and to provide exercise counselling for this rapidly increasing population.

## 5. Conclusions

Standardized medical history questioning makes it possible to functionally categorize young patients with CHD according to a specifically modified NYHA classification (NYHA-CHD). Indeed, the subgroups of NYHA-CHD class I, IIA and IIB significantly differed with regard to exercise capacity, tolerance and cardiorespiratory efficiency. It can thus be suggested that the adapted NYHA-CHD classification could help to refine the routine clinical evaluation of these patients also without complex testing procedures, possibly leading to a facilitated management and decision making for all clinicians and exercise professionals in charge of these patients. More data are needed to better investigate the impact of ventilatory-perfusion mismatch on functional limitation in these patients classified in the lower NYHA-CHD classes. However, maximal exercise testing should be regularly performed during patient follow-up, to objectively measure functional and aerobic capacity as well as cardiorespiratory efficiency, in order to monitor disease progression and provide cardiovascular risk stratification as well as individualized exercise prescription [31].

## Figures and Tables

**Table 1:** Functional evaluation of patients with congenital heart disease in different NYHA classes.

	NYHA I (*n* = 235)	NYHA IIA (*n* = 66)	NYHA IIB (*n* = 31)	I vs. IIA	I vs. IIB	IIA vs. IIB
**Age (years)**	13 (11.0–15.0)	14 (11.8–15.0)	13 (11.0–15.0)	0.290		
**Gender (male %)**	163 (70)	38 (57.6)	19 (61.3)	0.060	0.327	0.729
**BMI (kg/m^2^)**	19.3 ± 3.4	19.7 ± 2.3	18.4 ± 3.7	1.000	0.521	0.258
**BMI (percentiles)**	47.7 ± 29.7	49.3 ± 32.9	46.1 ± 32.2	0.933	0.955	0.881
**Resting HR (bpm)**	67 (60–75)	70 (63–81)	74 (67–82)	0.042	0.001	0.127
**HR max (bpm)**	190 (181–196)	176 (159–187)	162 (139–181)	<0.001	< 0.001	0.083
**HR max (% of predicted)**	91 (87–94)	86 (77–90)	79 (67–87)	<0.001	<0.001	0.054
**HR Reserve (bpm)**	120 (111–130)	105 (95–116)	88 (67–99)	<0.001	<0.001	0.023
**Exercise time (min:sec)**	11:54 ± 1:46	10:25 ± 1:56	8:34 ± 2:14	<0.001	<0.001	<0.001
**METs max**	16.8 (15.3–17.8)	13.9 (13.1–15.5)	11.0 (10.0–12.7)	<0.001	<0.001	<0.001
**VO_2_ peak (l/min)**	1.93 (1.55–2.46)	1.58 (1.23–1.85)	1.18 (0.91–1.44)	<0.001	<0.001	0.001
**VO_2_ peak (ml/kg/min)**	42.4 ± 7.8	31.9 ± 4.7	27.3 ± 5.0	<0.001	<0.001	0.010
**OUES (*n* = 319)**	1848 (1554–2290)	1479 (1276–1826)	1057 (867–1399)	<0.001	<0.001	0.001
**PETCO_2_ at anaerobic threshold (mmHg) (*n* = 321)**	37.8 (35.4–39.9)	33.5 (29.7–36.7)	28.7 (26.3–34.7)	<0.001	<0.001	0.079
**VE/VCO_2_ at anaerobic threshold (*n* = 321)**	26.8 (24.7–29.2)	31.0 (27.7–34.8)	34.8 (28.0–40.5)	<0.001	<0.001	0.129
**VE/VCO_2_ at peak exercise (*n* = 321)**	31.4 (28–33.5)	35.6 (28.9–42.1)	37.3 (31.4 – 45.1)	<0.001	<0.001	0.178
**SpO_2_ at rest (%) (*n* = 329)**	100 (99–100)	98 (96–100)	98 (94–100)	<0.001	<0.001	0.104
**Peak SpO_2_ (%) (*n* = 322)**	98 (97–99)	95 (91–98)	93 (87–98)	<0.001	<0.001	0.531

Table 1 shows the comparison of chronotropic response to exercise, functional and aerobic capacity, as well as cardiorespiratory efficiency, between adapted NYHA-CHD classes. HR, Heart Rate (maximal predicted HR: 220-years of age); HR Reserve, max-rest; METs max, maximum metabolic equivalents of task; VO_2_peak, peak oxygen consumption; OUES, oxygen uptake efficiency slope; PETCO_2_, partial pressure of end tidal CO_2_; VE/VCO_2_, ventilatory equivalent for CO_2_; SpO_2_, peripheral oxygen saturation. Mean value ± standard deviation or median with interquartile range (IQR) are shown when, respectively, normally or not-normally distributed.

**Table 2:** The proposed adapted NYHA-CHD classification for children with congenital heart disease.

		SYMPTOMS
**NYHA I**		Always asymptomatic. Physical activity and exercise do not cause fatigue, palpitations and dyspnea, even when performed at strenuous intensity or compared to their peers.
**NYHA II**	**IIA**	Asymptomatic for every-day life activities but show a slight limitation to competitive physical exercise among peers during leisure time activities and sports as well as at high intensity efforts.
	**IIB**	Slight limitation during physical activity. No symptoms at rest. Ordinary activities cause fatigue, palpitations or dyspnea.
**NYHA III**		Marked limitation in physical activity due to symptoms, even during less-than-ordinary activity. Comfortable only at rest.
**NYHA IV**		Severe limitations. Experiences symptoms even while at rest.

Patients can be classified based on the following standardized questions: (a) Do you feel you are limited during physical activities or exercise compared to your peers? (b) Would an external viewer notice some differences in your performance compared to your peers or teammates? (c) Does it occur that you feel any kind of functional limitation during strenuous physical activities or exercise?

## Data Availability

The data that support the findings of this study are available from the corresponding author upon reasonable request.

## Supplementary Materials

The following supporting information can be downloaded at: https://www.mdpi.com/article/10.3390/ijerph19105907/s1, Table S1: Prevalence of NYHA classes for each congenital heart disease (CHD).
