# Supplementary material for: Exercise Capacity and Cardiorespiratory Fitness in Children with Congenital Heart Diseases: A Proposal for an Adapted NYHA Classification
Source: Int J Environ Res Public Health. 2022 May 12;19(10):5907. doi: 10.3390/ijerph19105907 (PMC9141857; doi:10.3390/ijerph19105907)
Supplement: Supplementary file 1 [file ijerph-19-05907-s001.zip › ijerph-1680664-supplementary.pdf]

Supplementary Table S1. Prevalence of NYHA classes for each congenital heart disease (CHD).

CHD	NYHA I	NYHA IIA	NYHA IIB
Coarctation of the aorta (n=121)	114 (94,2%)	4 (3,3%)	3 (2,5%)
Tetralogy of Fallot (n=77)	51 (66,2%)	22 (28.6%)	4 (5.2%)
Functionally univentricular hearts with Fontan intervention (n=65)	17 (26.2%)	31 (47.7%)	17 (26.2%)
Transposition of the great arteries (n= 48)	42 (87.5%)	5 (10.4%)	1 (2.1%)
Complete atrio-ventricular septal defect (n=6)	5 (83.3%)	0	1 (16.7%)
Transposition of the great arteries with a ventricular septal defect (n=5)	2 (40%)	2 (40%)	1 (20%)
Congenitally corrected transposition of the great arteries (n=4)	1 (25%)	0	3 (75%)
Complex anomalies (n=4)	3 (75%)	1 (25%)	0
Partial atrio-ventricular septal defect (n=2)	0	1 (50%)	1 (50%)

Supplementary Table S1 shows the prevalence of the NYHA classes, according to the proposed adapted classification for children with congenital heart disease (NYHA-CHD I, IIA and IIB), among children with similar congenital heart disease.
